# Force Control Is Related to Low-Frequency Oscillations in Force and Surface EMG

**DOI:** 10.1371/journal.pone.0109202

**Published:** 2014-11-05

**Authors:** Hwasil Moon, Changki Kim, Minhyuk Kwon, Yen Ting Chen, Tanya Onushko, Neha Lodha, Evangelos A. Christou

**Affiliations:** 1 Department of Applied Physiology and Kinesiology, University of Florida, Gainesville, FL, United States of America; 2 Department of Physical Therapy, University of Florida, Gainesville, FL, United States of America; University of Alberta, Canada

## Abstract

Force variability during constant force tasks is directly related to oscillations below 0.5 Hz in force. However, it is unknown whether such oscillations exist in muscle activity. The purpose of this paper, therefore, was to determine whether oscillations below 0.5 Hz in force are evident in the activation of muscle. Fourteen young adults (21.07±2.76 years, 7 women) performed constant isometric force tasks at 5% and 30% MVC by abducting the left index finger. We recorded the force output from the index finger and surface EMG from the first dorsal interosseous (FDI) muscle and quantified the following outcomes: 1) variability of force using the SD of force; 2) power spectrum of force below 2 Hz; 3) EMG bursts; 4) power spectrum of EMG bursts below 2 Hz; and 5) power spectrum of the interference EMG from 10–300 Hz. The SD of force increased significantly from 5 to 30% MVC and this increase was significantly related to the increase in force oscillations below 0.5 Hz (*R* ^2^ = 0.82). For both force levels, the power spectrum for force and EMG burst was similar and contained most of the power from 0–0.5 Hz. Force and EMG burst oscillations below 0.5 Hz were highly coherent (coherence = 0.68). The increase in force oscillations below 0.5 Hz from 5 to 30% MVC was related to an increase in EMG burst oscillations below 0.5 Hz (*R* ^2^ = 0.51). Finally, there was a strong association between the increase in EMG burst oscillations below 0.5 Hz and the interference EMG from 35–60 Hz (*R* ^2^ = 0.95). In conclusion, this finding demonstrates that bursting of the EMG signal contains low-frequency oscillations below 0.5 Hz, which are associated with oscillations in force below 0.5 Hz.

## Introduction

Force control is essential in many activities of daily living. The inability to control force is quantified as increased force variability and has functional consequences such as diminished capacity to execute accurate movements [1], [2], [3]. Our recent findings [4], [5] suggest that force variability is directly related to oscillations in force below 0.5 Hz. Nonetheless, it is unknown whether the oscillations in force below 0.5 Hz are evident in muscle activity. The focus of this paper, therefore, is to determine whether the oscillations in force below 0.5 Hz are related to oscillations in muscle activity.

The force output comprises of specific oscillations. Previous studies have demonstrated that the variability of the force output is related to oscillations in force below 4 Hz [6], [7], [8]. Our recent findings, however, clearly demonstrate that oscillations in force below 0.5 Hz contribute significantly to force variability [4], [5]. This is demonstrated by a positive and strong association between force variability and force oscillations ∼0.2 Hz for both young and older adults. Force oscillations below 0.5 Hz likely originate from an oscillatory input to the motor neuron pool from higher centers as indicated from increased oscillations below 0.5 Hz by stroke individuals. Therefore, the importance of this study is to further our understanding on how the central nervous system oscillates multiple motor units to control the force output.

Muscle activity, as measured with electromyography (EMG), also comprises of specific oscillations. These oscillations are evident in single motor units and whole muscle activity. DeLuca and Erim [9], for example, have demonstrated that single motor units exhibit common low-frequency oscillations at about 1 Hz, termed common drive. At the whole muscle level, the interference (raw) EMG also contains oscillations anywhere from 10–300 Hz, with a peak around 150 Hz [10]. Recently, Yoshitake and Shinohara [11] examined whether the oscillations in force below 5 Hz are evident in muscle activity. They hypothesized that low-frequency oscillations in muscle activity must be related to bursting of muscle activation. They determined bursting of muscle activity by low-pass filtering the rectified EMG signal at 5 Hz. Interestingly, they found that force variability (rate of force fluctuations) was temporally related to the EMG burst signal. These findings indicate that the EMG burst can be used to identify the low-frequency oscillations in muscle activity that contribute to force variability. This study expands their findings by examining whether the modulation of EMG bursts is coherent to the oscillations in force below 0.5 Hz.

The purpose of our study, therefore, was to determine whether force oscillations below 0.5 Hz are related to oscillations in muscle activity. We hypothesized that bursting of the EMG signal will contain low-frequency oscillations below 0.5 Hz and are associated with the oscillations in force below 0.5 Hz.

## Methods

### Participants

Fourteen young adults (21.07±2.76 years, 7 women) volunteered to participate in this study. All subjects reported that they were healthy without any known neurological or orthopedic problems and right handed according to a standardized survey [12]. The Institutional Review Board at the University of Florida approved the procedures, and subjects provided written informed consent before participating in the study.

### Experimental protocol

We examined the ability of individuals to exert a constant force with the left index finger. Each subject participated in one experimental session that lasted less than an hour. Prior to the experimental session each subject was familiarized with the task and procedures of the study. The familiarization period included a verbal explanation of the task and 5 practice trials with a different force level and visual angle from the data collection task. After the familiarization period, each subject performed the following: 1) MVC with abduction of the index finger; 2) Constant isometric force control task with the index finger at two force levels (5 and 30% MVC – counterbalanced); 3) repetition of the MVC task.

### Experimental arrangement

#### Experimental setup and apparatus

**Figure 1:**
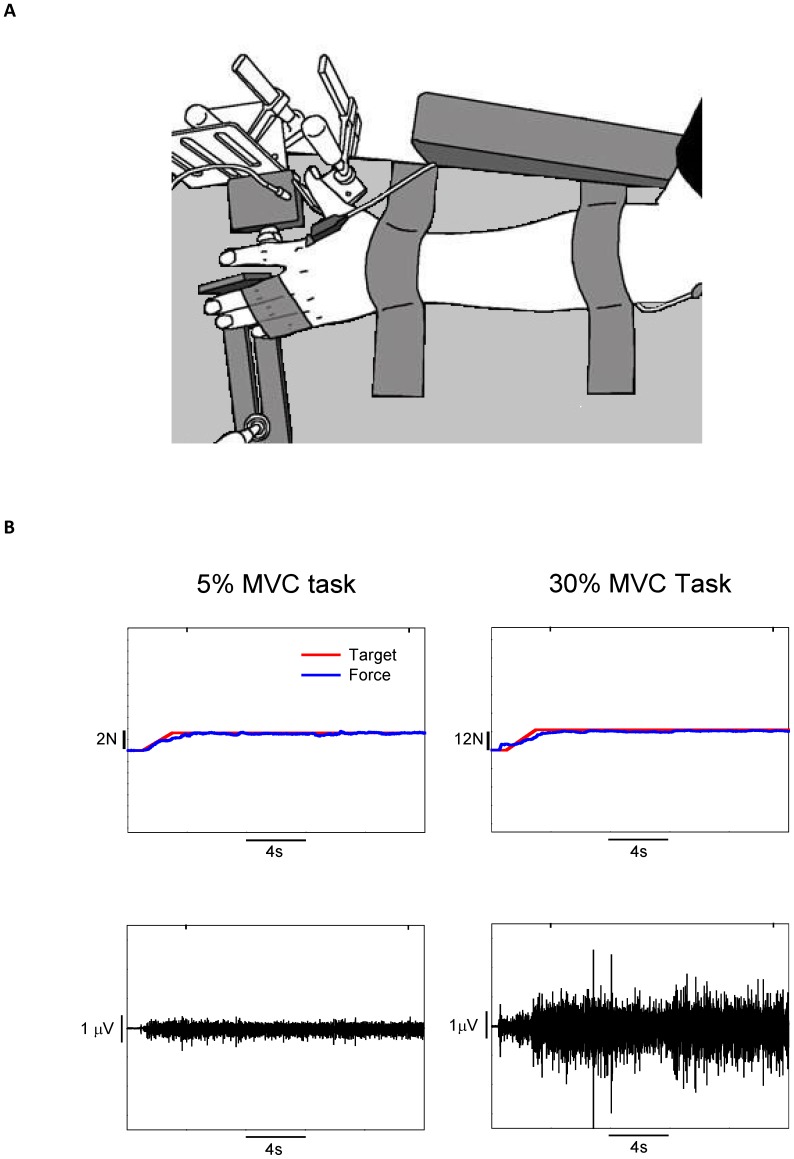
Experimental setup and sample force and EMG data. A) Top view of the experimental set up. We isolated the subjects’ wrist middle, ring and little fingers to allow only abduction of the index finger. The elbow was flexed at 90° and the shoulder was in 20° forward flexion and 30° abduction. B) Representative example of force and EMG signals at 5% and 30% MVC. The top row demonstrates the force task at 5 and 30% MVC, whereas the bottom row represents the interference EMG recorded at 5 and 30% MVC.

Subjects were seated comfortably in the upright position and faced a 32 inch monitor (SyncMaster 320MP-2, resolution: 1360×768 pixels, Samsung Electronics America, NJ, USA) located 1.65 m away at eye level. The monitor was used to display the force produced by the abduction of the index finger. All subjects affirmed that they could see the display clearly. The left shoulder was abducted at 45° and flexed to ∼90° at the elbow. The left forearm was pronated and immobilized by Velcro straps on a customized metal plate. Only the index finger was free to move (Figure 1A). This arrangement allowed the abduction of the index finger about the metacarpophalangeal joint in the horizontal plane. Abduction force is produced almost exclusively by the contraction of the first dorsal interosseus (FDI) muscle [13], [14]. We used the left hand in this study for two reasons: 1) it makes the task more novel to the subjects because the left hand is the non-dominant hand [10], [15]; 2) to compare the findings of this study with previous studies [16], [17], [18], [19].

#### Force measurement

The constant isometric force produced by the abduction of the index finger was recorded with a one-dimensional force transducer (Futek LRF400 -FSH00263, capacity: 5 lb; Futek Advanced Sensor Technology Inc. CA, USA). The force signal was sampled at 1 kHz with a Power 1401 A/D board (Cambridge Electronic Design, UK) and a NI-DAQ card (Model USB6251, National Instruments, Austin, TX, USA). The data were stored on a personal computer**.**

#### EMG measurement

The FDI muscle activity was recorded with a surface EMG electrode (Bagnoli TM, Single Differential, Delsys, Boston, MA, USA) that was taped on the skin distally to the innervation zone [20]. The recording electrode was placed in line with the muscle fibers. The reference electrode was placed over the lateral epicondyle of the humerus. The EMG signal was sampled at 1 kHz with a Power 1401 A/D board (Cambridge Electronic Design, UK) and a NI-DAQ card (Model USB6251, National Instruments, Austin, TX, USA). The EMG signal was amplified (×1,000) and [7], [15] high-pass filtered at 4 Hz (Bagnoli-16 Main Amplifier Unit, Delsys, Boston, MA, USA) and low-pass filtered at 500 Hz. EMG data were stored on a personal computer.

#### Maximum voluntary contraction (MVC)

Subjects increased abduction force of their index finger from baseline to maximum over a 3 s period and maintained their maximum force for 4 to 7 s. Each subject performed maximal trials until the maximum force of two trials was within 5% of each other. One minute of rest was given between trials. We used the average of the highest 3 s of force as the MVC. This procedure allowed for the identification of a more conservative MVC that reflects the capacity to maintain an isometric contraction. The MVC was repeated at the end of the experimental trials to determine whether muscle fatigue occurred.

#### Constant isometric force task

The target force was provided as a red horizontal line in the middle of the monitor and the force exerted by the subjects as a blue line progressing with time from left to right. The subjects were instructed to gradually push against the force transducer and increase their force to match the target force (red line) within 4 s (Figure 1B). When the target was reached, subjects were instructed to maintain their force (blue line) on the target as accurately and as consistently as possible. The visual angle for both force tasks was 0.02°. We used this visual feedback to eliminate the effects of visuomotor corrections on force control [7], [15]. Each subject performed two different submaximal isometric constant tasks (5% and 30% MVC). The order of the tasks was counterbalanced across subjects. For each task, subjects performed 5 trials and each trial lasted 20 s. Subjects had 20 s rest between trials and 3 mins rest between the two tasks. A custom-written program in Matlab (Math Works Inc., Natick, Massachusetts, USA) manipulated the targeted force-level and gain of visual feedback.

### Data analysis

Data were analyzed off-line using custom-written programs in Matlab (Math Works Inc., Natick, Massachusetts, USA). We analyzed all 5 trials recorded at each force level for each subject.

#### MVC

It was defined as the highest force exerted during abduction of the index finger. The EMG from the FDI muscle was also recorded and was used to normalize the EMG during the submaximal force contractions (5 and 30% MVC).

#### Force control

In this paper, we use force control interchangeably with standard deviation (SD) of force. In addition, we examined the power spectrum of force (PSD). A Fourier analysis was performed on the force signal [21]. The sampling frequency was 1 kHz. The window size was 15 s, which gave a resolution of 0.067 Hz. For statistical comparisons, the frequency data of the force signal were divided into four frequency bins: 0–0.5, 0.5–1.0, 1.0–1.5, and 1.5–2.0 Hz. These frequency bins, therefore, were based on the highest resolution of the Fourier analysis that could be accomplished with 15 s of force data. The dependent variable for the spectral analysis of the force signal was the relative power (%) in each data bin. The power was calculated as the power in each frequency bin relative to the total power of the force signal from 0–2 Hz.

#### Neural activation of the FDI

**Figure 2:**
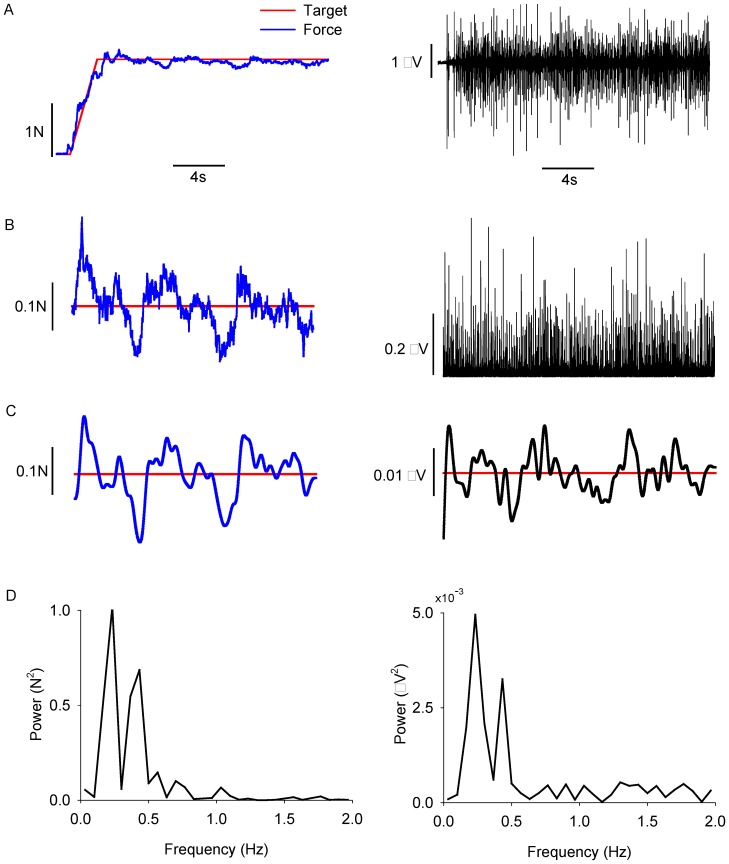
Quantification of low-frequency oscillations in force and muscle activity. A) The force task (left column) and its corresponding interference EMG (right column). B) The force signal (10–20 s; left column) and the corresponding rectified EMG signal used for analysis (right column). C) Both the force (left column) and the rectified EMG (right column) were low-pass filtered at 2 Hz. This low-pass filtering demonstrates the important frequencies in force and EMG bursting. D) The power spectrum density of the low-pass filtered force (left column) and low-pass rectified EMG (right column). Most power occurred below 0.5 Hz.

We examined the neural activation of the FDI with the interference EMG and the EMG burst activity. The interference EMG signal was the raw signal recorded from 10–300 Hz. The EMG burst signal was determined as follows: 1) The interference EMG was rectified; 2) The rectified EMG was smoothed by applying a second-order Butterworth digital filter with a cut-off frequency of 2 Hz (Figure 2B). 3) To determine the EMG burst oscillations, the 2 Hz low-pass filtered EMG signal was detrended. Detrending was performed using the detrend function in Matlab, which removes the best straight-line fit linear trend from the data. These procedures allowed us to examine the oscillations in the EMG signal below 2 Hz.

In addition, we examined the power spectrum (PSD) of the interference EMG and EMG burst signals. A Fourier analysis was performed on the two EMG signals [21]. The sampling frequency was 1 kHz. The window size was 15 s, which gave a resolution of 0.067 Hz. For statistical comparisons, the frequency data of the interference EMG signal were divided into five frequency bins: 10–35, 35–60, 60–100, 100–200, and 200–300 Hz. The frequency data of the EMG burst signal were divided into four frequency bins: 0–0.5, 0.5–1.0, 1.0–1.5, and 1.5–2.0 Hz. These frequency bins, therefore, were based on the highest resolution of the Fourier analysis that could be accomplished with 15 s of EMG data. The dependent variables for the spectral analysis of the EMG signals were the absolute and relative power (%) in each data bin. The relative power was calculated as the power in each frequency bin relative to the total power of the interference EMG signal from 10–300 Hz and of the EMG burst signal from 0–2 Hz.

### Statistical analysis

We used a dependent t-test to compare mean force and SD of force at 5 and 30% MVC. A two-factor repeated measures ANOVA (2 force levels x 4 frequency bands) was used to examine force and rectified EMG power across four frequency bands (0–0.5, 0.5–1, 1–1.5, 1.5–2 Hz). Similarly, we used a two-factor repeated measures ANOVA (2 force levels x 5 frequency bands) to compare the interference EMG power across five frequency bands (10–35, 35–60, 60–100, 100–200, 200–300 Hz). We used a linear regression model to determine the correlation (*R^2^*) between force oscillations below 0.5 Hz and EMG burst oscillations below 0.5 Hz. We used a stepwise multiple linear regression model to establish statistical models to predict the change in EMG burst oscillations from 5 to 30% MVC from the change of the interference EMG oscillations. The goodness-of-fit of the model was given by the squared multiple correlations (*R^2^*), Durbin Watson statistic (DW), and part correlation coefficients that demonstrate the unique contribution of each predictor to the criterion variable.

Analyses were performed with the IBM SPSS Statistics 21.0 statistical package (IBM Corp., Armonk, NY, USA). The alpha level for all statistical tests was 0.05. Data are reported as mean ± SD in the text and mean ± standard error of the mean (SEM) in the figures.

## Results

### Force control

**Figure 3:**
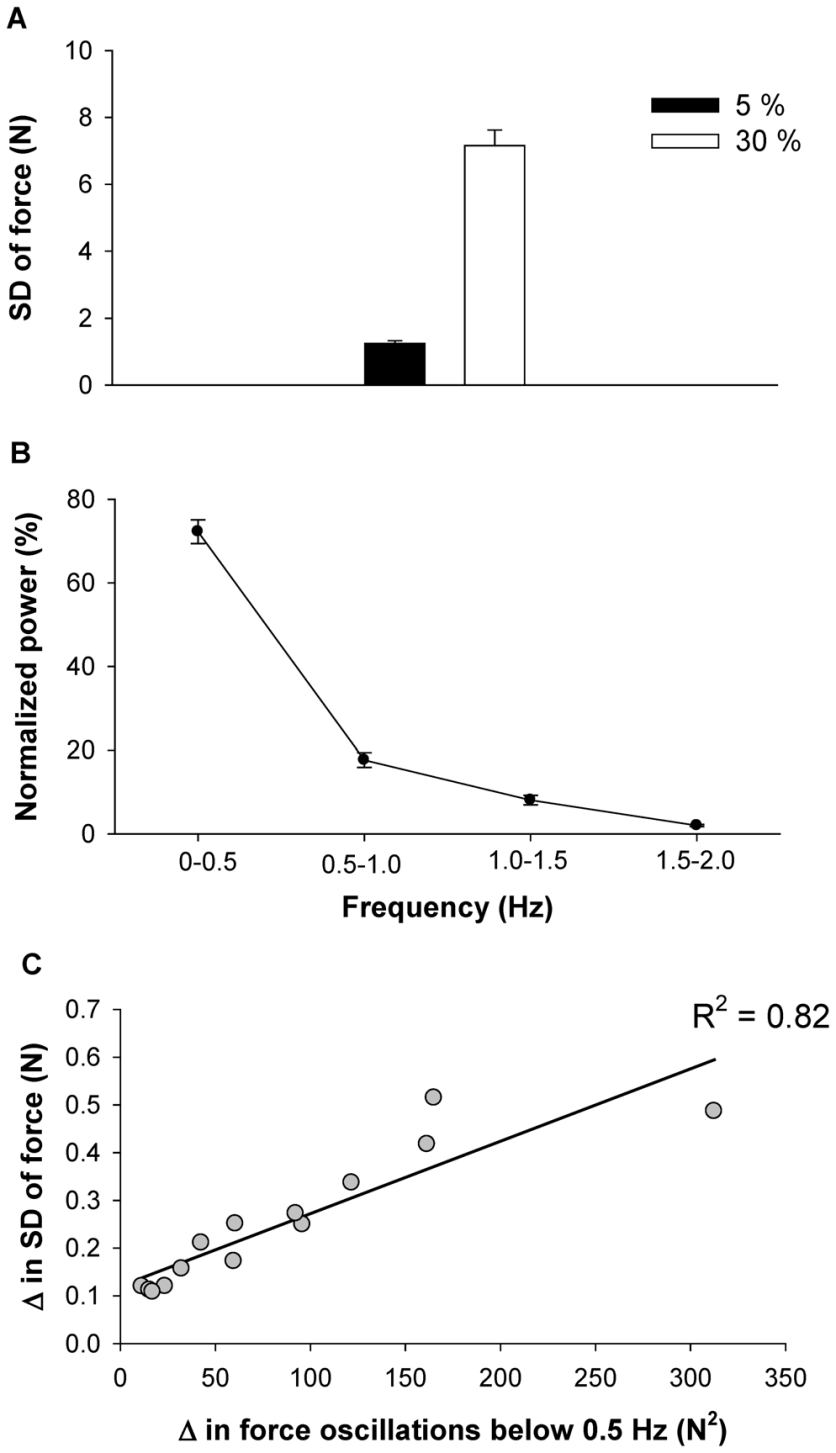
Force variability and oscillations in force. A) The SD of force for 5 and 30% MVC. As expected, the variability of force was greater with higher force. B) Normalized power spectrum density of force from 0–2 Hz. In this figure we present the average normalized power spectrum density because it was similar for the two force levels. The greatest power (∼75%) occurred below 0.5 Hz. C) The association between low-frequency oscillations of force and SD of force was strong (*R^2^* = 0.82). This indicates that ∼80% of force variability is due to the oscillations in force below 0.5 Hz.

The SD of force increased significantly from 5 to 30% MVC (*t* = 5.19, *P*<0.001; Figure 3A). The change in variability of force was ∼600%. Thus, the increase in SD of force with force level provided a good experimental model to determine whether increases in variability of force were related to force oscillations below 0.5 Hz, EMG burst oscillations below 0.5 Hz, and specific frequencies in the interference EMG.

The normalized power in force was similar for 5 and 30% MVC (frequency main effect; *F* = 32, *p*<0.05; Figure 3B). This figure demonstrates that oscillations in force primarily occurred from 0–0.5 Hz. The increase in SD of force from 5 to 30% MVC was strongly related with the increase in force oscillations from 0–0.5 Hz (*R* ^2^ = 0.82; Durbin Watson = 2.1; Figure 3C). This finding is in line with our previous work demonstrating that an increase in force variability is related to an increase in force oscillations from 0–0.5 Hz.

### Neural activation of the FDI

**Figure 4:**
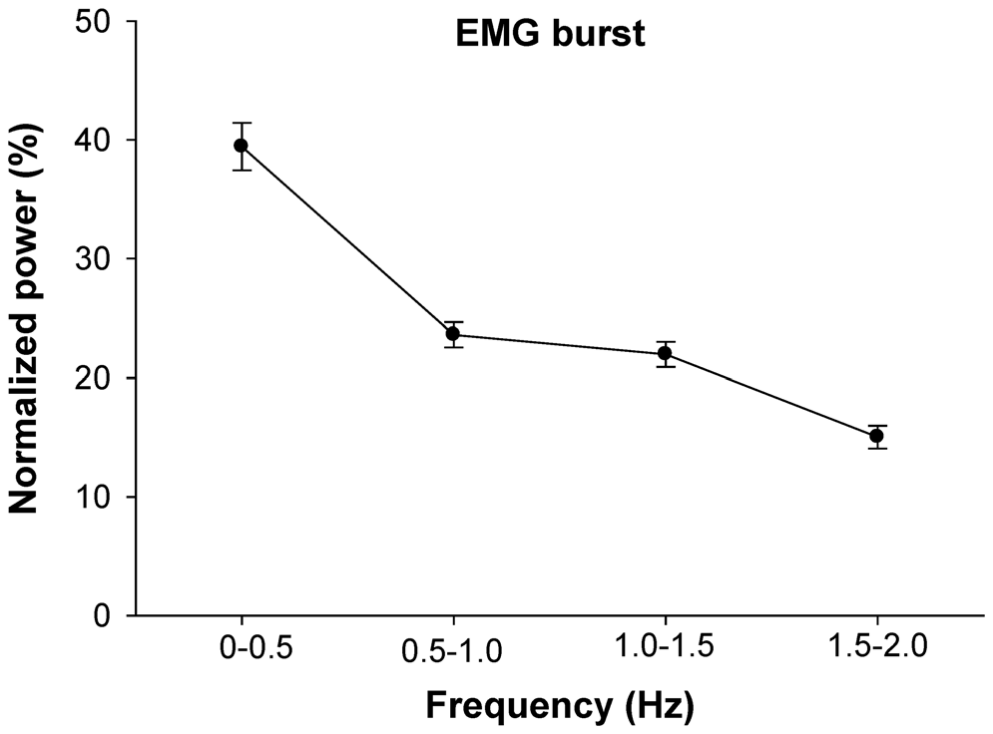
Normalized power spectrum density of low-pass rectified EMG power from 0–2 Hz. In this figure we present the average normalized power spectrum density because it was similar for the two force levels. The greatest power (∼40%) occurred below 0.5 Hz.

The EMG activity of the FDI increased significantly from 5 to 30% MVC (*t* = 5.19, *P*<0.001). The normalized power in EMG burst oscillations was similar for 5 and 30% MVC (frequency main effect; *F* = 32, *P*<0.05; Figure 4). This figure demonstrates that EMG burst oscillations primarily occurred from 0–0.5 Hz. In addition, the normalized power of the interference EMG was similar for 5 and 30% MVC (frequency main effect; *F* = 32, *P*<0.05) and most of the power occurred from 100–200 Hz.

### Oscillations in force and muscle activity

**Figure 5:**
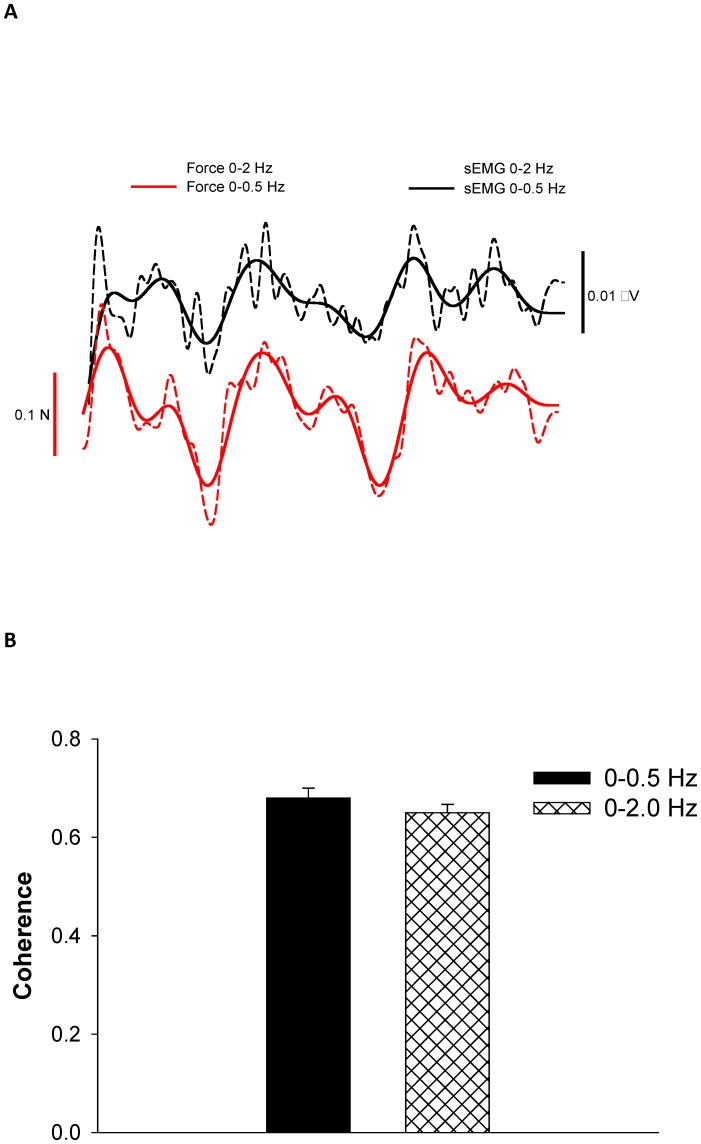
Coherence between the force and EMG burst oscillations. A) Representative example of a force and an EMG burst oscillations signal for 20 s. It is obvious that both signals exhibit common low-frequency oscillations. The dotted line represents low-pass filtered force and EMG burst at 2 Hz, whereas the solid line represents low-pass filtered force and EMG at 0.5 Hz. B) The overall coherence between the force and EMG burst oscillations when low-pass filtering the signals at 0.5 Hz and 2 Hz. The coherence for the two filtering procedures was similar, which indicates that low-pass filtering the force and EMG signals at 0.5 Hz captures well the synchrony between the two signals.

**Figure 6:**
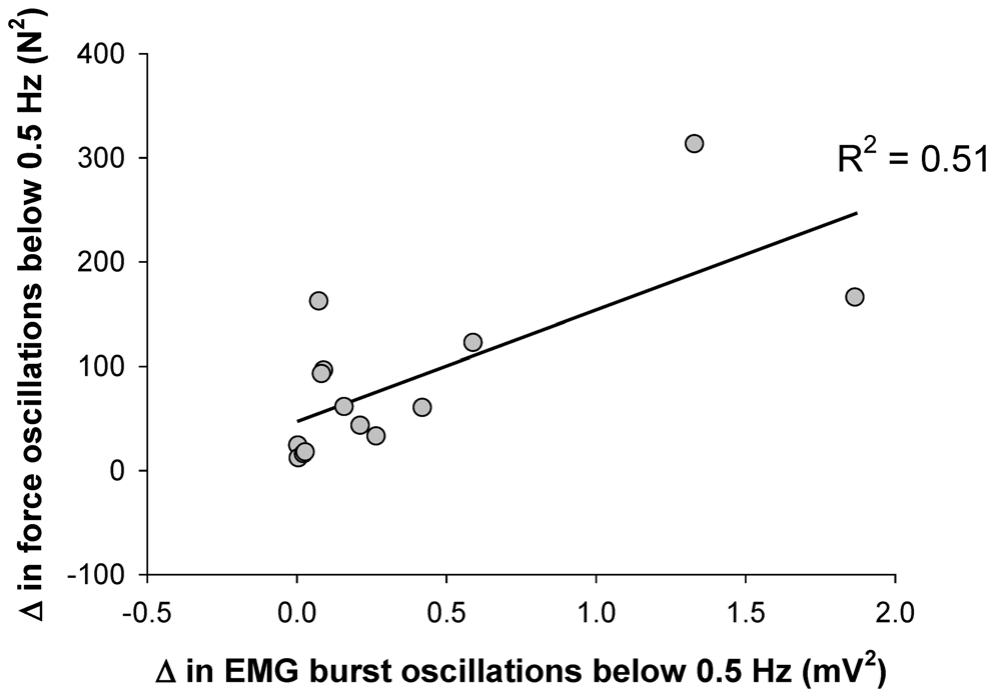
Changes (from 5 to 30% MVC) in force and EMG burst oscillations below 0.5 Hz. The association between changes in force oscillations below 0.5 Hz and changes in EMG burst oscillations below 0.5 Hz was moderate (*R^2^* = 0.51). This indicates that ∼50% of the force oscillations below 0.5 Hz are related to the EMG burst oscillations below 0.5 Hz.

Most of the power occurred from 0–0.5 Hz for force and EMG burst. We found that the oscillations in this frequency band were coherent for force and EMG burst (Figure 5A and B). Specifically, the coherence for force and EMG burst oscillations below 0.5 Hz was 0.68±0.1. The coherence for force and EMG burst oscillations below 2 Hz was 0.65±0.1. These findings demonstrate that common oscillations for the force and EMG burst signals occur at frequencies below 0.5 Hz. Furthermore, the increase in the force oscillations below 0.5 Hz from 5 to 30% MVC was related to an increase in EMG burst oscillations below 0.5 Hz (*R* ^2^ = 0.51; *DW* = 2.1; Figure 6).

**Figure 7:**
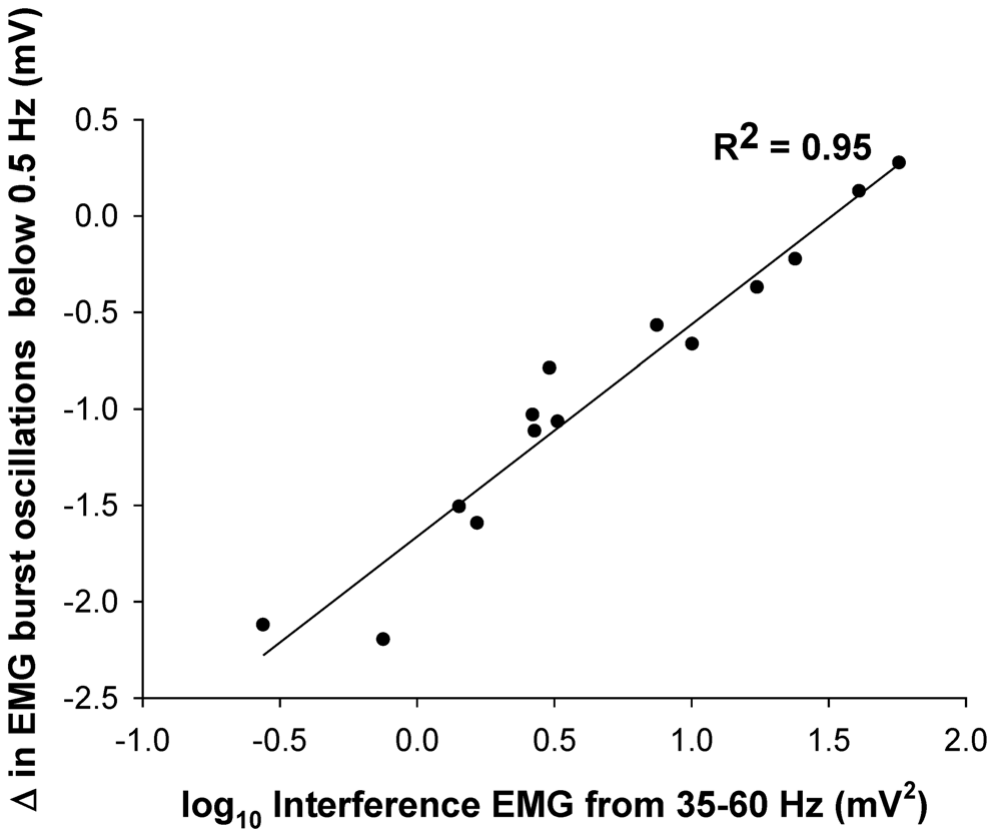
Changes (from 5 to 30% MVC) in EMG burst oscillations below 0.5 Hz and interference EMG. Based on the multiple regression model, the change in power from 35–60 Hz in the interference EMG strongly predicted the EMG burst oscillations below 0.5 Hz (*R^2^* = 0.95). This finding indicates that modulation of interference EMG from 35–60 Hz is strongly associated with EMG bursts below 0.5 Hz.

**Figure 8:**
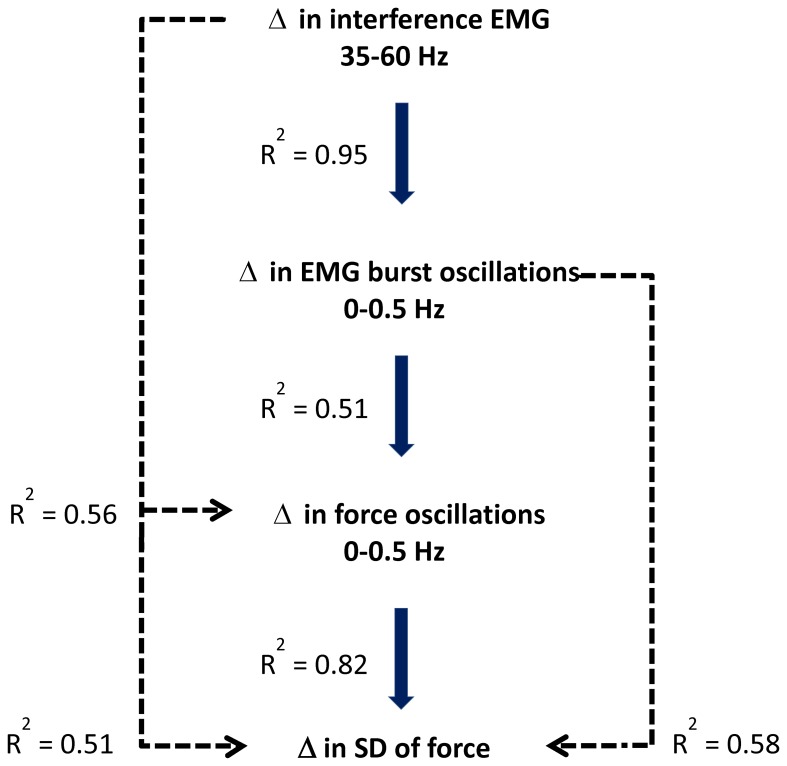
The associations between changes in force and muscle activity. We explain these associations starting at the bottom of the diagram. The change in variability of force (SD of force) from 5 to 30% MVC was strongly related to the change in force oscillations below 0.5 Hz (*R^2^* = 0.82). The change in force oscillations below 0.5 Hz was related to the change in EMG burst oscillations from 0–0.5 Hz (*R^2^* = 0.51). Interestingly, the change in EMG burst oscillations from 0–0.5 Hz was related to the change in power from 35–60 Hz in the interference EMG.

We examined the association between the EMG burst oscillations below 0.5 Hz and the power spectrum of the interference EMG. We logarithmically transformed the interference EMG because the association with EMG burst oscillations was non-linear. We performed this transformation by taking the log_10_ of the interference EMG for each band (10–35, 35–60, 60–100, 100–200, and 200–300 Hz). There was a significant and strong association between the increase in EMG burst oscillations below 0.5 Hz and the interference EMG from 35–60 Hz (*R* ^2^ = 0.95; *DW* = 2.1; Figure 7). Figure 8 diagrammatically summarizes all the associations between force and EMG variables.

## Discussion

In this paper we asked whether the oscillations in force below 0.5 Hz are evident in muscle activity. We demonstrate that such oscillations exist in muscle activity, manifest as bursts in EMG, and are coherent with force oscillations. Furthermore, we demonstrate that the EMG burst oscillations below 0.5 Hz strongly relate to the oscillations in the interference EMG from 35–60 Hz. These results provide novel evidence that the fundamental oscillations that control force are coherent with oscillations in muscle activity. These findings, therefore, significantly contribute to our understanding of the neuromuscular control of force during voluntary contractions.

### Muscle activity oscillations below 0.5 Hz

The most important finding in this study was the demonstration of coherence between force and EMG oscillations below 0.5 Hz. We were able to identify such oscillations by examining the bursting of muscle activity from surface EMG signals. Similar to a previous study [11], we identified the EMG bursts by rectifying the EMG signal and low-pass filtering it at 2 Hz. Our interest was oscillations below 0.5 Hz because we have demonstrated before [4], [5] that force control during steady contractions is associated with oscillations in force below 0.5 Hz. Typically, identification of EMG bursts is used to determine the magnitude of force a muscle exerts [22]. Our work and that of Yoshitake and Shinohara [11] demonstrates that the bursting of muscle activity contains information other than that of magnitude of force. Specifically, EMG bursting contains low-frequency oscillations (Figure 4) that are related to force variability (Figure 5 and 6) and thus may provide us with critical information about how the nervous system regulates force control during steady force contractions.

EMG bursts likely reflect the summed activity of recruited motor unit action potentials. This is demonstrated in a recent paper by Farina and colleagues [23]. Specifically, the force oscillations were directly related to the summed activity of 9 individual motor units across 400 ms. Therefore, the EMG bursts below 0.5 Hz likely reflect a fundamental way the nervous system modulates the recruited motor units. This is important because this modulation is a strategy by the CNS to solve the abundant degrees of freedom (motor units) by grouping them to activate at a specific frequency (for an extensive discussion/review on this issue see Latash 2012). The question that arises is: “Why do we need the rectified EMG signal to see the modulation of muscle activity below 0.5 Hz?” This information is not readily available in the interference EMG for the following reasons: 1) the recorded interference EMG signal is typically high-pass filtered at 10–20 Hz and thus such information is significantly attenuated [24]. This high-pass filtering is essential to remove movement artifact from the EMG signal [11],[22],[24]. 2) The interference EMG is dominated by higher frequency power (100–200 Hz), which relates to the shape of the action potential [10], [24]. Nonetheless, the interference EMG contains information about the modulation of the active motor units from 10–100 Hz [10], [24], [25]. Interestingly, our results demonstrate that the EMG burst oscillations below 0.5 Hz are strongly correlated with the modulation of the interference EMG from 35–60 Hz (Figure 7). This would suggest that when the nervous system groups more motor unit action potentials at 35–60 Hz the EMG burst oscillations below 0.5 Hz also increase. Thus, force control would be impaired when the EMG signal contains more power from 35–60 Hz. Indeed, our previous studies demonstrate that the impaired ability of older adults to control force is related to greater power from 35–60 Hz in the interference EMG signal [26], [27]. In conclusion, these findings demonstrate that the 0.5 Hz oscillations in force are related to oscillations in muscle activity, evident as oscillations in the EMG bursting at 0.5 Hz and EMG interference signal from 35–60 Hz.

### Force oscillations below 0.5 Hz

Our results demonstrate that the force oscillations below 0.5 Hz are strongly related to force variability. Specifically, oscillations in force ∼0.2 Hz can explain about 80% of force variability. This result is consistent with our current findings in young and older adults [4] and stroke and healthy adults [5]. For example, the study by Fox et al. [4] indicated that increases in force variability with visual gain and aging were strongly associated with oscillations in force ∼0.2 Hz [4]. Furthermore, when chronic stroke individuals performed constant force tasks with the hand (gripping) they exhibited greater variability and force oscillations ∼0.2 Hz with their paretic arm compared with healthy individuals [5]. The oscillations ∼0.2 Hz are likely related to oscillatory drives from higher centers [28]. This hypothesis is derived primarily from two findings: 1) Changes in visual gain influence force oscillations ∼0.2 Hz. Because brain structures are responsible for the processing of visual information, it is likely that the modulation of 0.2 Hz oscillations in force occurs at higher centers [29], [30]. 2) The paretic arm of individuals that suffered a stroke exhibits higher power at 0.2 Hz compared with their non-paretic arm [5]. Because stroke affects brain structures, it is likely that the source of the oscillations in force ∼0.2 Hz occurs at higher centers. Regardless of the exact source of the ∼0.2 Hz oscillation in force, our findings link the variability of force to the activation of muscle.

### Limitations

Our results are limited to the surface EMG signal. It is not clear whether such low-frequency oscillation exists in single motor units (although see [23], [28], [31]). Nonetheless, individual motor units may act differently than groups of motor units, which is what we record with surface EMG signals. In addition, because our subjects were limited to healthy young adults, it is not clear whether our findings will generalize to other populations with greater force variability such as children [32], older adults [4], [27], or adults with neurological disorders [5]. Future studies, therefore, are needed to examine the association of EMG burst oscillations to force variability in other populations.

In summary, we provide evidence that oscillations below 0.5 Hz occur in muscle activity, manifest as EMG bursts, and are coherent with the oscillations in force that contribute the most to force variability. This is important because the findings from this paper link the variability of the motor output with the activation of muscle at frequencies below 0.5 Hz. Such findings are the first in the motor control literature that demonstrate an association between force and EMG oscillations below 0.5 Hz. These results, therefore, enhance our understanding of how the nervous system controls force during steady contractions.

## Supporting Information

Data S1 (XLSX)
